# Realizing nurses’ full potential

**DOI:** 10.2471/BLT.15.030915

**Published:** 2015-09-01

## Abstract

Sheila Tlou tells Fiona Fleck about the innovative approaches to the HIV epidemic she helped to develop by blending her talents as professor of nursing, civil society activist and director of a theatre company.

Q: What drew you to public health?

A: Originally I wanted to study languages, do drama and end up in Hollywood. But when I was interviewed by the Ministry of Education for a scholarship, I was told: “In Botswana we don’t eat languages, we are a developing country. We need doctors and nurses and the only scholarship available is in the health sciences”. I was so disappointed. They gave me three study options: in Uganda, Zambia or Ethiopia for public health, which I chose only because of the handsome guys on the brochure. However, a scholarship to study nursing in [the United States of] America came up and I ended up in New Orleans.

Q: How did you get involved in the HIV response in Botswana in the early days?

A: I started teaching at the university of Botswana in 1980, and my interest was in women’s health, so I became involved in gender activism and women’s health issues, specifically ageing, and my subsequent doctoral dissertation was on menopause. At a regional women’s health meeting in Uganda in 1984, I met Noerine Kaleeba, a Ugandan physiotherapist, who told us about the discrimination she faced in her country after her husband was diagnosed with HIV [human immunodeficiency virus]. Noerine was one of the first people to fight discrimination faced by people living with HIV. When the first case of AIDS [acquired immunodeficiency syndrome] was found in Botswana, and people started to become infected with HIV, I was determined to be part of stopping the discrimination. HIV is mainly transmitted by sex – something done by everyone – so I had a strong conviction that there should be no stigma or discrimination. I thought that within 10 years the epidemic would be over and I would go back to working on ageing and menopause.

Q: Countries are pledging to end the HIV epidemic by 2030, as one of the targets of the new Sustainable Development Goals to be adopted by countries at the United Nations General Assembly this month. Is this a realistic target?

A: It is a realistic target. Our confidence is based on success in achieving previous targets. For example, the target of providing 15 million people with life-saving treatment was reached in March this year, nine months ahead of the deadline. As part of our fast-track strategy, UNAIDS and our partners aim to end HIV infection as a public threat by achieving the 90–90–90 targets to keep people alive, reduce new infections and ensure zero discrimination. The targets are that 90% of people living with HIV know their status, 90% of them are on treatment and 90% have viral suppression.

Q: What are the particular risks to women’s health and how did you raise greater awareness of this?

A: Women are more vulnerable to HIV infection due to gender inequality in our societies. I met with several African women involved in the AIDS response at the International AIDS conference in Stockholm in 1988. We formed the Society for Women and AIDS in Africa and set up national chapters in our countries. I founded the Society for Women and AIDS in Africa Conference in Botswana (SWAABO). The idea was to make every woman in each of our countries aware of the risks, so that they could take measures to prevent HIV infection for themselves, families and communities, and to care for people living with AIDS without stigma or discrimination. Two years later, in 1990, I organized the first SWAABO conference. Women living with HIV from all over the continent came and, for the first time, many people in Botswana saw the face of HIV.

Q: You and your colleagues pioneered community-based approaches to HIV prevention, care and support in Botswana. How did you do it?

A: We started with young people in schools. Some teachers were not trained for this, so we put on theatre sketches to educate them and trained them to educate their peers and their pupils in all aspects of HIV and AIDS. At that time, there were no drugs for HIV infection, so our focus was on prevention, management, care and support. We supported women with HIV, for example, by explaining how the infection was transmitted, especially from mother to child. By this time many women were dying, leaving orphans. We engaged lawyers to help them make living wills. We lobbied the Botswana government to launch a home-based care pilot programme based on a successful one that had been started by a community in northern Botswana, and had the participation and financial support of the community, and the government was able to roll it out across the country.

Q: So you managed to incorporate your passion for drama in the HIV response?

A: Yes. Part of the home-based care approach was developed in response to what women said they needed at home. They had to negotiate safer sex with their husbands and so SWAABO did role playing with them to help them with the negotiating skills they needed to develop. I was also quite involved in amateur theatre and co-directed the University of Botswana travelling theatre company; we toured the country putting on performances to educate people about HIV. That‘s how I realized drama could change people’s attitudes to HIV.

Q: As health minister you helped to pioneer nurse-based prescription of antiretroviral (ARV) medicines in Botswana, how did it start?

A: Botswana was advanced in primary health care, financed by the diamond extraction industry, well before the advent of HIV. We had health-care facilities every 5 km, staffed by nurses. We didn’t have a medical school and so we had very few doctors – most of them were foreign. So, when responding to the HIV epidemic, it was about working with the people on the ground – who had always been the backbone of our health system – and they were nurses.

“We managed to show the Western world that we could provide ARVs universally in a low-income country.”

Q: How did you train nurses to prescribe ARVs?

A: When ARVs first came onto the market in the 1990s, people said it would be impossible to roll these out in Africa because there weren’t enough doctors and the health infrastructure was weak in many places. I was determined to show that we could do it. In 2001, when Botswana started rolling out treatment, while I was teaching at the university, I worked with the Ministry of Health and the Botswana Harvard AIDS Institute Partnership to develop a training course called KITSO (knowledge, innovation and training shall overcome AIDS). In Setswana KITSO means *knowledge*. Through the KITSO course we trained all health personnel, including nurses, to prescribe ARVs. It was so successful that within 4 years 90% of people in need were on ARV treatment and being followed up. So we managed to show the Western world that we could provide ARVs universally in a low-income country and this is also something we could give back to the Western world.

Q: You also helped to develop an innovative approach to HIV testing. How?

A: Many people were not coming forward for testing. People who suspected they had the infection feared a positive result was a death sentence, they feared the stigma and didn’t realize that treatment was available and could lengthen their lives. So we met with all our partners and civil society. Our idea was that each health facility in Botswana should have a person who was living with HIV to counsel and offer HIV testing to anyone who came in, no matter what they came for. That person would say: “I know my status. I am living with HIV and I have access to drugs. Do you want to know your status?” We called it the “opt-out routine offer of HIV testing”. At the time, WHO and much of the global public health community said it was “home grown” and not applicable in other countries. But when the international community heard about our work at the International AIDS Conference in Toronto in 2006 and we published our findings in *PloS Medicine* in 2006, our approach won international acclaim and WHO used this to develop *Guidelines on provider-initiated HIV testing and counselling in health facilities*. Botswana pioneered that approach and gave this to the world.

Q: Nurses account for an estimated 60–70% of the additional health workforce needed to achieve universal health coverage. How can developing countries build their nurse workforces?

A: Some countries need to upgrade skills that their nurses already have so that, for example, they can prescribe and follow-up clients living with HIV. Countries should also implement the WHO *Global code of practice on the international recruitment of health personnel*. I served on the committee that developed this and I am firmly convinced that countries need to train more health personnel and take measures to retain them, so that they benefit their countries of origin.

“The first nurses to be trained in Botswana were princesses and the children of upper-class families.”

Q: How did Botswana move away from the traditional model of nurses as the assistants of physicians to their autonomous role today?

A: The first nurses to be trained in Botswana were princesses and the children of upper-class families, thus nurses were always well respected and became autonomous because they were responsible for all aspects of health care, including planning. After independence in 1966, institutes of health sciences assumed responsibility for training nurses. A bachelor’s degree programme was introduced at the University of Botswana in 1978, and later master’s degree programmes and further training at PhD level. And so Botswana’s nurses never saw themselves as physician’s assistants but as colleagues working under the leadership of either the nurse practitioner or the medical doctor.

Q: During your term as health minister you rolled out almost universal access to ARVs with nurse-led prescribing. What are the factors for an empowered nursing workforce?

A: The nurses who prescribed ARVs in Botswana were registered nurses, nurse practitioners, nurses with diplomas and degrees (bachelors, masters and PhDs) whose pre-service and in-service training incorporated substantial knowledge on HIV. Moreover, some were doing original research on the impact of HIV on communities in Botswana. Although they were better paid than other nurses, they were not so much motivated by high salaries as by having the ability to transform their patients’ lives, often from a state of hopelessness to one in which they could go back to work and lead productive lives again.

